# Innervation of a Prefabricated Flap: A New Experimental Model

**DOI:** 10.1155/2014/549819

**Published:** 2014-07-24

**Authors:** Marco Romeo, Giuseppe Cuccia, Shan Shan Qiu, Stefania Raimondo, Stefano Geuna, Bernardo Hontanilla

**Affiliations:** ^1^University Clinic of Navarra, Plastic Surgery Unit, Avenida Pio XII 36, 30001 Pamplona, Spain; ^2^Fatebenefratelli Buccheri La Ferla Hospital, Plastic Surgery Unit, 90123 Palermo, Italy; ^3^Department of Clinical and Biological Sciences, University of Turin, Regione Gonzole 10, Orbassano, 10043 Turin, Italy

## Abstract

*Introduction*. Flap innervation by neoaxonogenesis is a promising field of investigation. The authors evaluated the possibility of innervating an acellular collagen scaffold as component of a potential prefabricated flap.* Materials and Methods*. Collagen matrix sheets were implanted around the femoral bundle of a murine model to produce two flaps on proximal and distal nerve stumps based on a flow-through model. After thirty days, nerve regeneration and integration into the collagen matrix were evaluated. The specimens were microscopically analyzed to study Schwann cell colonization and axonal integration with the matrix. Axonal count and density were assessed and statistically evaluated.* Results*. Qualitative structural and ultrastructural evaluation indicated integration, with axonal fibers merged within the collagen matrix, along with a newly formed vascular network on the proximal flap. Wallerian degeneration occurred inside the distal chamber. Axonal count and density did not show statistically significant differences between the nerve inside the proximal flap and the control side.* Conclusions*. Innervation of an acellular matrix can be obtained by direct nerve stump implantation. The flow-through system was relatively easy to build and reliable to provide adequate blood supply. The collagen scaffold may be a promising support or further studies of preinnervated microsurgical flaps.

## 1. Introduction

Tissue engineering is raising great expectations in regenerative medicine. It aims to understand the basic mechanism of tissue growth to bridge from an* ex vivo* to an* in vivo* setting aimed at facilitating clinical applications [1] to repair, maintain, and eventually improve tissue/organ function.

Bitto et al. [2] and Messina et al. [3] investigated the integration of a nerve stump in a AV-loop prefabricated flap as source of stimulation for target tissues (fat or muscle). In this study we explored the possibility of performing innervation of an acellular dermal matrix (using the* in vivo* flow-through model tested by Tanaka et al. [4]) by creating a sealed vascularized flap including a peripheral nerve.

## 2. Material and Methods

Sixteen male Wistar rats (average weight 300 gr.) were operated at CIFA (Centre for Applied Pharmacological Investigation, University of Navarra, Pamplona, Spain); power analysis was performed to ensure statistic consistence of the samples. Experiments, care, and treatment of the animals were consistent with national and EU laws and policies (EEC Council Directive 86/609, OJL 358, December 12, 1987). All operations were carried out under general anaesthesia using an intraperitoneal injection of Ketamine (75 mg/kg) and Xylazina (10 mg/kg); each animal was kept in a single cage and euthanized at the end of observation period with an overdose of anesthesia.

### 2.1. Flap Construction

**Figure 1:**
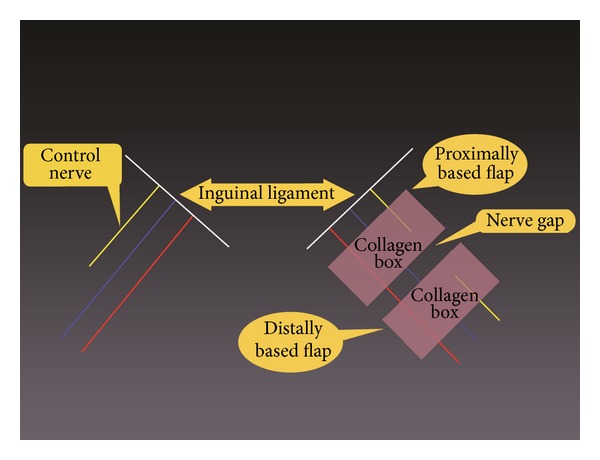
Schematic representation of study concept and flaps construction. On the right limb (left on the image) the femoral nerve was cut and left to physiological growth evolution. On the left limb (right on the image) the femoral nerve was cut and the proximal and distal stumps were buried in the collagen sheets (pink rectangles) and sealed with the silicon layer. The vascular bundle (blue and red lines) was freely dissected from collateral vessels to maintain the flow-through model.

Femoral bundles were exposed on both limbs. The right thigh nerve was dissected from artery and vein, transected, and used as a control group for normal femoral nerve morphology at day zero and after one month (Figure 1).

**Figure 2:**
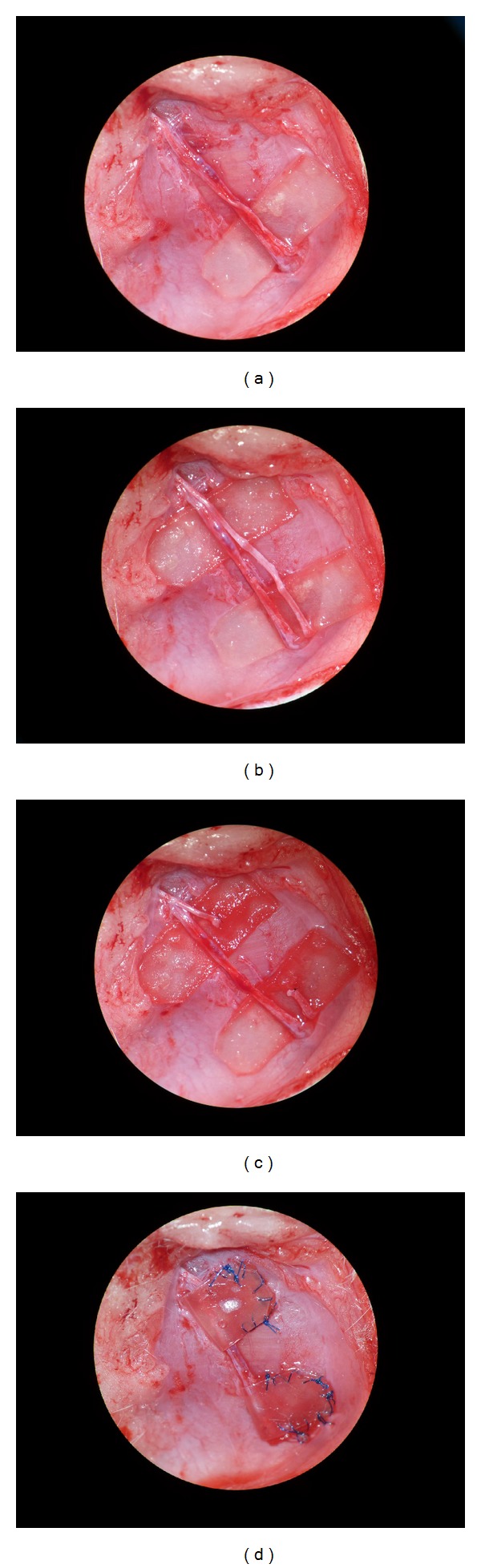
Construction phases of the two flaps. (a) The pedicle is harvested, the nerve separated, and the first piece of Integra is placed. (b) The two layers of collagen are placed. (c) The nerve is cut and the two stumps are secured onto the collagen. (d) The boxes are sealed with nylon suture and anchored to the underlying muscle.

On the left limb the artery and vein were kept intact and freely dissected from the collateral vessels to maintain the flow-through model. The nerve was cut leaving a 20 mm gap between the two ends and the collagen scaffold sheets (Integra, Plainsboro, New Jersey, USA) were folded around the bundle to create two 15 × 15 mm flaps. The nerve was stitched to the collagen matrix with 9/0 nylon to prevent accidental extrusion; a 15 mm gap was left between the flaps. The outer silicon layer was left to isolate the flaps from external contaminations and closed with running 6/0 Ethilon. A further stich was placed to anchor each flap to surrounding tissues to avoid accidental malposition, rotation, flaps contact, and torsion of the pedicle (Figure 2).

### 2.2. Histological and Biological Analysis

At day zero, the control nerve was cut for morphological evaluation.

After thirty days, the patency test was performed by clamping the artery, emptying a tract of the vessel and reestablishing the flow, the same was done with the vein to confirm adequate outflow, and the flaps were then removed. All sixteen rats had a 2 mm portion of the stump of the control nerve cut and studied.

The rats were randomly divided in two groups to study the flaps in the left thigh: in eight rats, the outer silicone layer only was removed and whole collagen-vessels-nerve complex was embedded in paraffin and processed for histological and immunohistochemical analysis. Specimens were stained with Masson trichrome method (Bio-Optica, Milan, Italy). For immunohistochemistry, the following antibodies were used: anti-S100 (polyclonal, rabbit, dilution 1 : 800, Sigma, St. Louis, MO), for detecting Schwann cells, and anti-beta-tubulin (monoclonal, mouse, dilution 1 : 1,000, Sigma, St. Louis, MO), for detecting axons. In the other eight rats, both flaps were opened and the nerve stump was carefully dissected and harvested, embedded in resin, and processed for high-resolution light and electron microscopy and histomorphometry. For light microscopy and histomorphometry, semithin sections (2.5 *μ*m) were stained by toluidine blue and examined in a DM4000B microscope equipped with a DFC320 digital camera (Leica Microsystems, Wetzlar, Germany).

For electron microscopy, ultrathin sections (70 nm) were stained with uranyl acetate and lead citrate and examined in a JEM-1010 transmission electron microscope (JEOL, Tokyo, Japan). Axonal count was performed with ImageJ (National Institute of Health, USA) by analyzing standardized images of sections of the nerves at 300 dpi of resolution. Axonal density was performed by simple pixel/mm conversion delimitating an area in the core of the nerve of known size [5].

All data collected from axonal count/density groups were analyzed with a Pearson test to verify correlation between overall number of axons per section and density before and after the observation period. The Fischer test was used to observe any significant difference among the proximal, distal, and control groups evaluating the number and density of axons/mm^2^.

## 3. Results

No rats died and no complications were encountered apart from a silicon layer extrusion. The authors avoided this problem using the inguinal fat pad to bury the flap. At day thirty, before removing the flaps, patency test was 100% positive for distal perfusion and no vascular failure was recorded.

### 3.1. Microscopy and Immunohistochemistry Analysis

**Figure 3:**
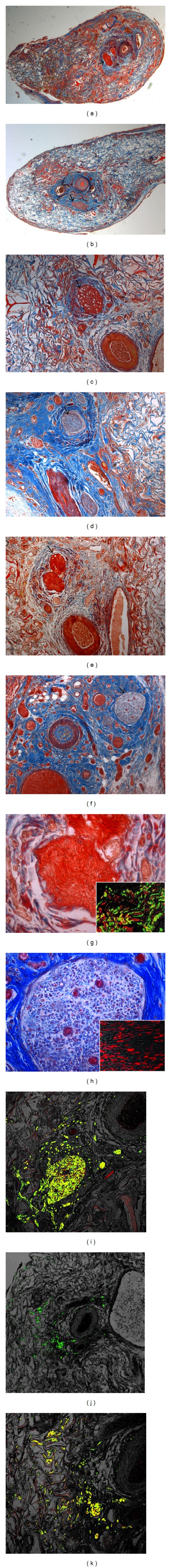
Light microscopy (trichrome staining) and immunofluorescence (S-100-red and beta-tubulin-green) of paraffin sections from collagen chambers with proximal nerve stump ((a), (c), (e), (g), (i), (j), and (k)) and distal nerve stump ((b), (d), (f), and (h)). The arrows point to the nerve located close to the vascular bundle. Original magnification: (a), (b) = 25x; (c), (d) = 100x; (e), (f) = 200x; (g), (h) = 400x; box in (g) and (h) = 600x; (i), (k) = 100x; (j) = 80x.

In both proximal and distal flaps, trichrome staining has been performed and images at different magnifications are presented in Figure 3. Low magnifications (Figures 3(a) and 3(b)) allowed visualizing the entire flap and suggested the formation of more abundant tissue with a richer vascular network in the proximal flap (Figure 3(a)) than in the distal one (Figure 3(b)). The collagen matrix (blue stained) had been colonized by abundant new tissue especially in the proximal flap. Within the neurovascular bundle, the femoral nerve (arrows in Figures 3(a)–3(f)) can be observed close to the vessels.

Higher magnification of femoral nerve showed a different morphology between proximal (Figure 3(g)) and distal (Figure 3(h)) flap. Axons (red stained) were more abundant in the proximal part than in the distal. Immunohistochemistry after axonal and glial marker employment revealed that, in the proximal stump, both axons (green) and Schwann cells (red) were still present (box in Figure 3(g)). By contrast, in the distal stump only glial cell markers, but not axonal markers, were detected (box in Figure 3(h)). This indicated the occurrence of selective axonal involution typical of distal Wallerian degeneration. Moreover in the proximal flap, 30 days after surgery several sprouted fibers are detected in the collagen matrix around the neurovascular bundle (Figures 3(i)–3(k)).

**Figure 4:**
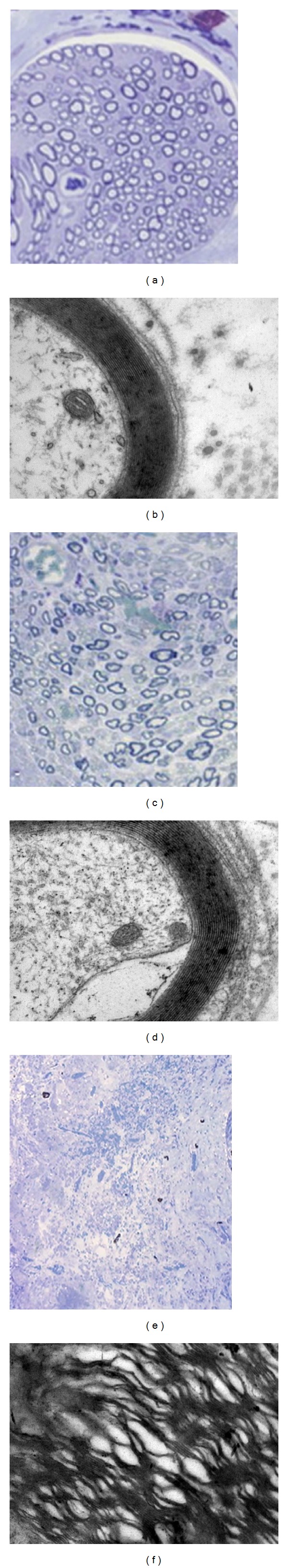
High resolution light microscopy ((a), (c), and (e)) and electron microscopy ((b), (d), and (f)) of control femoral nerve ((a), (b)), proximal nerve stump ((c), (d)), and distal nerve stump ((e), (f)) buried in the artificial flap. Original magnification: (a), (c), and (e) = 400x; (b), (d), and (f) = 80,000x.

High resolution light and electron microscopy (Figure 4) confirmed that the proximal nerve stump (Figures 4(c) and 4(d)) was organized similarly to the control nerve (Figures 4(a) and 4(b)). Wallerian degeneration occurred in the distally based stump (Figures 4(e) and 4(f)), where abundant myelin destruction can be observed.

**Figure 5:**
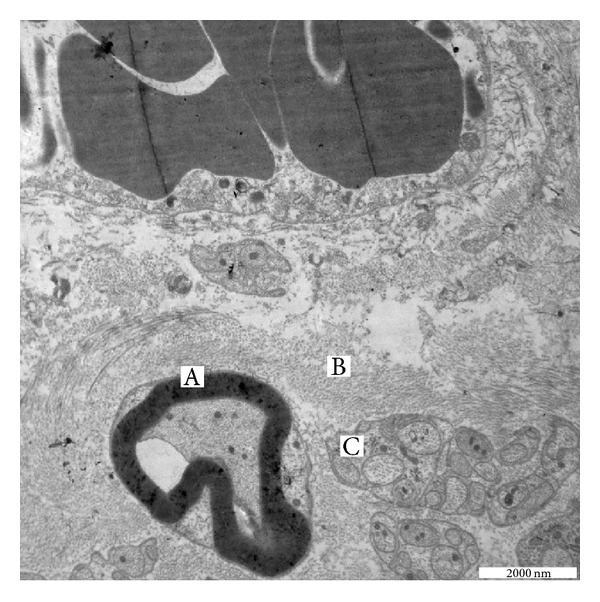
Electron microscopy image of the peripheral collagen layer. (A) Newly formed myelinated fibers. (B) Collagen fibres. (C) Nerve sprouting of unmyelinated fibers. A vessel can be seen on the top center of the image.

Proximal flap's wall images with electron microscopy showed the presence of random nerve fibers and vessels merged into the collagen matrix (Figure 5).

### 3.2. Nerve Count and Density

Histomorphometry (number of myelinated fibers and axonal density) has been performed on control and proximal and distal femoral nerve immediately (Day 0) and 30 days (Day 30) after surgery as reported in Table 1. No significant (*P* > 0.05) differences have been observed between control and proximal femoral nerve, both at day 0 and day 30. The distal stump at day 30 showed no countable amount of axons.

Pearson's test gave a weak (*r* = 0.42) correlation between average axons number and density of axon/mm^2^for the control nerve and a mild positive correlation for the proximally based nerve (*r* = 0.66).

## 4. Discussion

### 4.1. Vascular Preconditioning

An adequate vascular support has been shown to be a basic condition to promote nerve outgrowth in the matrix. Neoangiogenesis is a time related process; therefore it is important to allow sufficient time to develop a new vascular network and nerve regeneration with it.

According to Pribaz and Fine [6], a prefabricated flap requires an 8-week maturation of a vascular pedicle inside a volume of tissue to promote neovascularization. Although this definition is still widely accepted, other authors have published similar results with shorter time of neovascularization. Muller et al. [7] achieved vascularization of a synthetic resorbable scaffold based on the epigastric bundle of rat within three weeks, while Wang et al. [8] demonstrated angiogenesis after just two weeks. MacLeod et al. [9] found vascularity on a dermal matrix after two weeks on a rat model based on the epigastric artery (similar to our model) and successfully implanted it as a microvascular free flap. Houle and Neumeister brought these principles into the clinical setting, creating a completely new free flap [10].

According to Tanaka et al. [4] the flow-through model is a simpler but equally reliable system to A-V microsurgical loop flap [11] and might represent an adequate model for further studies. The good sealing achieved with the outer silicon layer and subsequent capsular formation guarantee a sufficiently isolated scenario without external influences, that might be ideal for testing growth factors within the flap as bioreactor.

### 4.2. Matrix Innervation

Other authors have previously investigated the possibility of innervation of an artificial construct with different aim or kind of scaffold. Suuronen et al. [12] were able to innervate a tissue-engineered cornea adding neural growth factor to promote SC (Schwann cells) progression. Parenteau-Bareil et al. [13] affirmed that a three-dimensional tissue-engineered nervous system model can be used to promote axonal migration by SC stimulation through a connective tissue (due to its porous and reabsorbable characteristics). Fukushima et al. [14] successfully tested a honeycomb collagen scaffold to reinnervate a transected spinal cord.

Similar to neoangiogenesis, innervation of artificial flaps can either follow an extrinsic pathway or ensue from intrinsic innervation. In the former condition, the axonal colonization of the flap is limited by the absence of SC that guide axons and support formation of functionally active nerve fibers. Schwann cells express surface proteins and elaborate growth factors that lead to the extension of the regenerating axonal ends [15, 16].

We hypothesized that, to overcome this limitation, intrinsic preinnervation and flap vascular preconditioning should be sought. We tested preinnervation by using proximal and distal nerve stumps. As expected Wallerian degeneration of the axons occurred in the distally based flap [17].

On the other hand, the proximal construct showed nerve integration within the collagen matrix. Immunohistochemistry (Figure 3) and electron microscopy (Figure 5) demonstrated that from the main stump several fibers invaded the collagen flap with a vascular sprout inside the collagen matrix next to it. An ordinate pattern of Schwann cells and the absence of scarring exclude the possibility of confusing this pattern with a newly formed neuroma [18–20].

## 5. Conclusion

Various prefabricated flaps have been described in the past. Preinnervated vascularized flaps represent a promising new class of bioengineered* in vivo* constructs that might improve the effectiveness of advanced tissue engineering and organ regeneration.

## Figures and Tables

**Table 1:** Histomorphometrical evaluation of myelinated fibers and axonal density in control and proximal femoral nerve.

	Day 0		Day 30	
	Number of fibers	Axonal density	Number of fibers	Axonal density
Control nerve				
Mean ± SEM	1467 ± 203	8596 ± 253	1663 ± 229	8215 ± 305
Proximal nerve				
Mean ± SEM	1564 ± 158	8639 ± 258	1700 ± 152	8883 ± 259
